# Overexpression of miR-149 attenuates opioid-related perturbations in neural stem cell fates and serves as a translational biomarker for infants with prenatal opioid exposure

**DOI:** 10.1371/journal.pone.0345640

**Published:** 2026-03-31

**Authors:** Rhea E. Sullivan, Claire J. Miller, Quinn Ahrens, Brittany J. Fronheiser, Fumiyuki C. Gardner, Emma Raich, Sara Mills-Huffnagle, Christiana Oji-Mmuo, Steven D. Hicks

**Affiliations:** 1 Department of Pediatrics, Penn State College of Medicine, Hershey, Pennsylvania, United States of America; 2 Department of Advanced Medicine, Hospital of the University of Pennsylvania, Philadelphia, Pennsylvania, United States of America; 3 Department of Medicine, Penn State College of Medicine, Hershey, Pennsylvania, United States of America; 4 Department of Neuroscience and Experimental Therapeutics, Penn State College of Medicine, Hershey, Pennsylvania, United States of America; 5 Department of Pediatrics, Dell Medical School at the University of Texas, Austin, Texas, United States of America; Purdue University, UNITED STATES OF AMERICA

## Abstract

There is currently no biomarker to predict the maximum morphine dose (MMD) for newborns experiencing withdrawal from chronic prenatal opioid exposure (POE). This is due, in part, to a lack of understanding about how the developing brain is altered by chronic opioid exposure and withdrawal on a molecular level. We previously developed a human induced pluripotent stem cell-derived model of POE and withdrawal to examine the impact on neural progenitor cell fates. Here, we leveraged our model to investigate the role of two microRNAs implicated in both neural stem cell differentiation and opioid signaling: miR-149 and miR-23b. Further, we asked if these microRNAs were related to the need for morphine treatment and MMD in the saliva of infants with POE. Levels of miR-149 (One-way ANOVA, F = 34.18, *p* < 0.0001), but not miR-23b (One-way ANOVA, *p* = 0.14), were significantly decreased in human neural progenitors after chronic morphine exposure (Tukey’s, adj. *p* = 0.004), and decreased further in those that underwent withdrawal compared to vehicle exposed controls (Tukey’s, adj. *p* < 0.0001). The relevance of miR-149 to neonates experiencing withdrawal after POE was confirmed by decreased salivary levels of miR-149 compared to levels in healthy infants 24–96 hours after birth (n = 56, 28 unexposed and 28 infants with POE) (Mann-Whitney U, *p* < 0.0001). Stratifying infants with POE by need for pharmacotherapy revealed a further decrease in levels of miR-149 in infants that required treatment (One-way ANOVA, *p* < 0.0001). In a hierarchical linear regression model utilizing infant demographic factors, addition of miR-149 levels in neonatal saliva improved performance for predicting the MMD necessary for symptom control (R = 0.673, *p* = 0.002). These results indicate the potential relevance of miR-149 levels in infants with prenatal opioid exposure. Validation in larger cohorts is necessary.

## Introduction

Precipitated by the ongoing opioid epidemic, generations of infants have been affected by chronic prenatal opioid exposure (POE). [1] Between 2008 and 2022, over half a million children in the United States have been hospitalized at birth with withdrawal symptoms secondary to POE. [2] In some states, neonatal opioid withdrawal syndrome (NOWS) incidence is as high as 23 per 1000 births. [3] Hospital costs for NOWS increased 3-fold between 2012 and 2016, totaling $2.5 billion in 2016 alone. Medicaid is the largest payer for NOWS-related costs, covering 83.3% of all NOWS-related costs. [3] This issue remains a prominent public health issue, as there is accumulating evidence that chronic opioid exposure and withdrawal may have negative long-term effects on child health [1,4–6].

An infant with POE is at risk for NOWS at birth, which is characterized by central nervous system manifestations of withdrawal, such as tremors, a high-pitched cry, sleep disturbances, increased muscle tone, myoclonic jerks, irritability and seizures. [7,8] In a retrospective cohort study, infants treated for NOWS (n = 87) with either methadone, buprenorphine, or morphine were more likely to have developmental delays and a lower intelligence quotient (IQ) at two years of age compared to a normative sample. [9] Behavioral outcomes in children with POE may include increased rates of attention deficit hyperactivity disorder (ADHD). In another study, children with POE (ages 6–14) had more symptoms associated with ADHD and autism spectrum disorder compared to a control group (n = 228), after controlling for sociodemographic factors and medical history. [10] Of this cohort, 8% of children had been treated for strabismus by age 3, which recapitulated similar adverse visual outcomes identified in other cohorts of infants with POE. [11,12] Despite such reports, the mechanism for how chronic opioid exposure and withdrawal affects the developing brain remains unknown.

Alterations in proliferation and differentiation of neural stem cells are thought to be a major point of convergence in neurodevelopmental disorders. [13] In fact, 70% of genes identified by the Simon’s Foundation Autism Research Initiative as drivers of a neurodevelopmental syndrome are associated with cell proliferation or differentiation. [13] In addition to germline mutations, environmental impacts during pregnancy such as substance use, affect neural stem cells. Preliminary studies in mouse embryonic stem cells have shown that morphine exposure decreases neuronal differentiation and opioid receptor expression. [14] This provides a strong rationale for understanding the molecular mechanisms of how chronic opioid exposure and withdrawal may impact neurodevelopmental processes involving human neural stem cells.

Our incomplete understanding of how chronic opioid exposure affects the developing human brain makes it difficult to predict which infants with POE will experience NOWS. The current gold-standard diagnostic paradigm relies on withdrawal severity scoring (Finnegan Neonatal Abstinence Scoring System (FNASS)) for treatment initiation and guidance. [15] However, the FNASS does not predict the morphine dose required to control an infant’s withdrawal symptoms. As a result, days to weeks can be spent titrating morphine pharmacotherapy to identify the optimal maintenance dose. [16] During this time, the infant may experience unmanaged discomfort, or receive more cumulative morphine exposure than necessary. The current approach may also prolong hospital length of stay and healthcare costs. [17] Currently, there are no objective biomarkers that inform of accurate morphine dosing in infants with NOWS.

MicroRNAs (miRNAs) are short, non-coding transcripts that influence human brain development [18–20] through post-transcriptional regulation of neurodevelopmental gene targets. miRNA expression in neural stem cells can be impacted by environmental toxins. [21] Opioid-responsive miRNAs have been implicated in neurodevelopmental processes. [22] For example, miR-149-3p has been shown to be a regulator of human neural progenitor differentiation. [23] Additionally, miR-149-3p is also predicted to target protein kinase C subunit α (PRKCA), which is an important player in the morphine withdrawal response. [24,25] Putative transcript targets of miR-149-3p relevant to neurodevelopment include synaptic Ras GTPase activating protein 1 (*SYNGAP1*) and methylation CpG binding protein 2 (*MECP2*). [25] Single nucleotide polymorphisms (SNPs) in both of these genes lead to devastating neurodevelopmental disorders due to deficits in neural stem cell differentiation. [26,27] Additionally, miR-149-3p is a known regulator of toll-like receptor 4 (TLR4). TLR4 has been shown to bind morphine, fentanyl, and oxycodone by interacting with the lipopolysaccharide-binding pocket of myeloid differentiation-2 (MD-2). [28] Opioid binding subsequently stimulated nuclear factor kappa-light-chain-enhancer (NFκB) for release of pro-inflammatory cytokines. [28]

Interestingly, saliva has the highest detectable number of miRNAs across 12 biofluids. [29] Additionally, saliva contains the third highest concentration of miRNAs out of 12 human biofluids, after seminal fluid and breast milk. [29] Saliva miRNAs are mostly derived from exosomes, allowing for their stabilization in fluctuating pH levels and protection from enzymatic degradation. [30] Our previously published work demonstrates that salivary miRNA profiles reflect those of cerebrospinal fluid in patients with traumatic brain injury. [31] We have previously shown in large cohort studies that salivary miRNA profiles can be used to differentiate children with autism, [32] and to differentiate adolescents with mild traumatic brain injury. [33] Saliva-based biomarkers are ideal for neonates, as they can be measured using non-invasive collection. [34] Until now, the roles of miRNAs that are both opioid-responsive and regulate neurodevelopmental processes have not been investigated in the context of NOWS brain pathophysiology.

We previously developed a novel model of chronic prenatal opioid exposure and withdrawal using human induced pluripotent stem cell (iPSC)-derived midbrain neural progenitors. [35] Strengths of this model system include the ability to control human neural stem maturation during chronic opioid exposure and withdrawal with the same endogenous cues that occur *in vivo*. We found that prolonged morphine exposure decreased the proportion of cells that were neuronal nuclear antigen (NEUN)^+^, a marker of mature neurons, in favor of an increased Nestin (NES)^+^ positive pool, a marker of progenitors. These results were not related to differences in cytotoxicity, levels of opioid receptor expression, or enhanced neural stem cell proliferation.

This study sought to determine if miRNAs mediate perturbations in human neural stem cell differentiation induced by chronic opioid exposure and withdrawal. We identified 4 miRNAs (let-7a-5p [36–39], miR-192-5p [25,36,40], miR-23b-3p [36,41], miR-149-3p [23,25,42,43]) that are known to: 1) be expressed in neural stem cells; 2) have important roles in neural differentiation; and 3) have either predicted or experimentally validated opioid-related mRNA targets. We hypothesized that at least one of these miRNAs would be altered by opioid exposure and/or withdrawal in neural stem cells, simulated by washout. Further, we posited that transfection of an opioid-responsive miRNA would alter cell fate commitments in human induced pluripotent stem cell (iPSC)-derived neural stem cells. In addition, we examined the utility of miRNAs as translational biomarkers in a longitudinal cohort study of infants with/without POE. We hypothesized that miRNAs that regulate neural stem cell fate following opioid exposure would display similar perturbations in the saliva of infants with POE and aid prediction of maximal morphine dose needed for symptom control. Identification of miRNAs that respond to opioid exposure and opioid withdrawal could provide mechanistic insights and yield an objective measure to aid clinical management of POE.

## Methods

### Human iPSC-derived midbrain neural progenitor culture

A healthy human iPSC line (hiPSC), SCTi003-A (female donor), was purchased from the manufacturer (STEMCELL Technologies, Vancouver) (hPSCreg: SCTi003-A) and maintained on lactose dehydrogenase elevating virus (LDEV)-free Geltrex (Gibco, NY) and mTesR PLUS (STEMCELL Technologies, Vancouver), as previously described.(44) Stock ampules were frozen in liquid nitrogen in cryopreservation media. Only cells with < 10 passages were used for neural induction and downstream experiments. Neural induction via SMAD inhibition and midbrain patterning using recombinant sonic hedgehog (rhSHH) were conducted according to the longer neural induction and patterning methods previously described. [35] All pluripotency, neural induction, midbrain patterning, and neuronal/glial neuronal differentiation products were purchased from STEMCELL Technologies. [35] This line has been extensively characterized for lack of genetic aberrations (karyotype abnormalities or sites of common copy-number variations). [44] Further, characterizations of these human iPSC-derived midbrain neural progenitors verified that the expected markers of regional and cellular identity were present, along with the expression of important opioid receptors and downstream withdrawal-related machinery. [35]

### Defining each cell culture treatment group in modeling POE

Three treatment conditions of midbrain neural progenitors were compared as previously described. [35] Briefly, in Condition 1, hiPSC-derived midbrain neural progenitors which had completed regional patterning were further cultured for 5 days in STEMDiff™ Midbrain Neuronal Differentiation Media + 200 ng/mL rhSHH with an equal volume of 1X phosphate buffered saline (PBS) as a vehicle control to morphine-exposed cells. This media does not stimulate differentiation, but rather promotes regional midbrain patterning. On day *in vitro* (DIV) 42, neuronal and glial differentiation was begun by changing the media to STEMdiff™ Midbrain Maturation Media, and media changes were made daily until DIV47. STEMdiff™ Midbrain Maturation Media allows neural progenitors to differentiate to terminal cell types (glia vs. neurons). Condition 1 represents the developing human brain without any opioid exposures. For Condition 2, midbrain neural progenitors were cultured as described above with the presence of 10 μM morphine sulfate [35,45,46] during both midbrain progenitor patterning and early neuronal and glial differentiation stage. This concentration of morphine was selected based on: 1) previously published morphine exposure studies [41,45,46], and 2) decreased culture time in monolayer relative to organoids cultures, which can be grown for >9 months at physiologic concentrations. Prior morphine dose-response curves in studies in human and mouse neural stem cells have strong phenotype readouts at this concentration, as well [45,47].

On day DIV47, morphine washout occurred, with morphine replacement occurring immediately after washout. [35] Media changes with morphine exposure continued daily until completion of neuronal and glial differentiation on DIV56. Condition 2 represents the developing brain with both POE during earlier developmental processes (i.e., regional patterning) *in utero*, and continued morphine exposure throughout neuronal maturation. Condition 3 is the same as Condition 2, however morphine washout occurs on DIV47 without replacement. This final condition represents the developing brain with chronic opioid exposure, though only experiences morphine withdrawal during the differentiation process.

### miRNA and mRNA quantification from hiPSC-derived midbrain neural progenitor cells

Twenty-four hours after morphine withdrawal (DIV48), neural progenitors were harvested and total RNA was extracted using the miRNeasy kit (Qiagen, Germantown). To identify when opioid-responsive miRNA levels would be altered after withdrawal, neural progenitors were harvested over a time course of 5, 12, 24, 48, and 96 hours. The chosen time for harvesting after withdrawal (24 hours) was selected due to detection of opioid-responsive miRNAs whose levels changed at 24 hours after withdrawal, but not at other time points after withdrawal. The 24 hour time point falls within known withdrawal symptom onset of NOWS infants (0–96 hours). [8] Poly-adenylation and mature miRNA first strand synthesis was completed using the MystiCq® miRNA cDNA synthesis kit (Sigma, Allentown). All primers for miRNA quantitative polymerase chain reaction (qPCR) were proprietary MystiCq® system primers (Sigma, Allentown), which have been experimentally validated by the manufacturer and by the author. Small nucleolar RNA C/D box 44 (SNORD44) was used as an internal control. MiRNA qPCR was completed using the MystiCq® Universal Reverse Primer (Sigma, Allentown) and MystiCq® miRNA SYBER Green (Sigma, Allentown). mRNA first strand synthesis was completed using Superscript II™ First Strand Synthesis System for reverse transcription polymerase chain reaction (RT-PCR) (Invitrogen, Waltham). To measure level changes in predicted targets of candidate miRNAs, PowerUP™ SYBER Green was used (Invitrogen, Waltham). DNA primers for putative miRNA targets were designed using experimentally validated sequences supplied by the Harvard Primer Bank (Integrated DNA Technologies, Coralville). All sequences are listed in S1 Table. Glyceraldehyde 3-phosphate dehydrogenase (GAPDH) was used as an internal control for mRNA target qPCR. All qPCR was repeated in technical triplicate on a QuantStudio™ 5 Real-Time PCR System machine. The ΔΔCt method was used to calculate changes in transcript levels relative to an internal control.

### miRNA-mRNA target selection

Putative mRNA targets of candidate miRNAs whose levels were significantly altered by morphine exposure or morphine withdrawal were selected by searching miRDB for neurodevelopmental or opioid-related targets with a target prediction score of 80 or above, or if the candidate miRNA had experimental evidence of targeting a known mRNA. miRDB target prediction scores are measures of confidence that a given mRNA transcript is a target for a given miRNA. Based on computational target prediction algorithms as described by miRDB, a predicted target with a score greater than 80 is most likely to be real. [25] From this list, we chose 5 putative targets with the highest possible target scores with known roles in neural stem differentiation or opioid signaling. Based off these parameters, the following mRNA transcripts were selected as putative targets for qPCR relative expression analysis: delta like non-canonical Notch ligand 1 (*DLK1*, miR-149 score = 91), protein kinase C alpha subunit (*PRKCA*, miR-149 score = 84), methyl CpG binding protein 2 (*MECP2,* miR-149 score = 86), and synaptic Ras GTPase-activating protein 1 (*SYNGAP1*, miR-149 score = 99). Two candidate miRNAs with regulatory roles in neurodevelopment, let-7a-5p and miR-23b-3p, have previously been experimentally validated as direct regulators of the mu opioid receptor transcript by binding to its 3’ untranslated region (UTR). [37,48]

### Biotinylated miRNA mimic transfection via electroporation

Synthetic double stranded miRNA mimics and scramble sequences with sense strand 3’ biotinylation were designed as previously described. [49] Double stranded miRNA mimics have been shown to have increased incorporation into Argonaute proteins relative to single stranded miRNA mimics. [49] This is due to the endogenous RNA-induced silencing complex (RISC) preferentially loading double stranded miRNA duplexes. [50,51] *C. elegans* miRNA cel-miR-243-3p has no homology to human genes, and was used as a negative control scramble sequence. All sequences for mimics are listed in S1 Table. The timeline for transfection experiments is as follows: On DIV37 according to our previously published culture model [35], 4 x 10^6^ hiPSC-derived neural progenitors were plated in a 12-well plate coated in poly-ornithine laminin in STEMDiff Midbrain Differentiation Media + 200 ng/mL rhSHH. Culture media was changed daily until DIV42. On DIV42, cells in each well were electroporated with 200 pmol of biotin-labeled duplexes at 100 V using the Ingenio® EZporator® Electroporation System (Millipore Sigma, Burlington). This low ratio of duplexes to cells has been previously identified to be ideal for studying direct consequences of miRNA binding to mRNA targets, and not effects due to changes in transcription factor targets. [49] Electroporated cells were re-plated on polyornithine laminin coated plates in STEMdiff™ Midbrain Maturation Media, in the presence or absence of 10 μM morphine sulfate. On DIV47, all conditions experienced washout by washing the well three times with 1 x PBS. Media without morphine was replaced for cells in Condition #3.

### Confirmation of miRNA:mRNA binding using hybrid Argonaute immunoprecipitations and biotinylated miRNA duplex pulldown

On DIV48 (24 hours after morphine withdrawal), cells were harvested in hypotonic lysis buffer containing 5 mM KCl, 1.5 mM MgCl_2_, 10 mM Tris-Cl pH 7.5, 5 mM DTT, 1% NP-40, 30 U/mL SUPERase In (Ambion, Austin) and 1X Complete Mini Protease inhibitor (Roche, Basal). [49] Cell debris was removed by centrifugation (10,000 x g for 2 minutes at 4°C). In order to enrich for miRNA:mRNA complexes bound to within the Argonaute 2 (AGO2) complex, AGO2 was first precipitated using the Dynabeads™ Protein G Immunoprecipitation Kit (Invitrogen, Waltham) and a mouse monoclonal antibody to AGO2, according to manufacturer’s instructions. The enriched fraction was brought to a final concentration of 1 M NaCl. myOne C1 Dynabeads (Invitrogen, Waltham) were blocked in 1 μg/μl of bovine serum albumin and 1 μg/μl of yeast tRNA (Invitrogen, Waltham) for 30 minutes at room temperature. Next, magnetically tagged streptavidin myOne C1 Dynabeads were incubated with the AGO2-enriched fraction for 30 minutes at room temperature. Washes were performed in accordance with manufacturer’s instructions and then separated on a magnet. Complexes of streptavidin-immobilized miRNA mimic and mRNA targets were resuspended in 1 M NaCl. Total RNA was extracted using the miRNeasy Micro kit (Qiagen, Germantown). First strand synthesis of mRNA targets was performed using the SuperScript II™ First Strand Synthesis System. Primers for putative targets were designed using experimentally validated sequences from the Harvard Primer Bank. All samples were run in technical triplicate.

### LIVE/DEAD cytotoxicity assay

To assess if the transfection of biotinylated miRNA mimic or scramble affected susceptibility to cell death secondary to morphine exposure or withdrawal, the LIVE/DEAD™ Viability/Cytotoxicity Kit (Invitrogen™, Waltham) was used and performed in accordance to the manufacturer’s protocol, for 3 biological replicates, 3 cell conditions, and the presence of biotinylated miRNA mimic/scramble control. LIVE/DEAD™ staining was performed 24 hours after morphine withdrawal. Results were quantified according to the microplate reader protocol listed within the manufacturer’s instructions.

### Quantitative immunocytochemistry

Immunostaining, image analysis, and statistics were performed as previously described. [35] Briefly, on DIV56 differentiated cell types were fixed in chilled 4% paraformaldehyde for 10 minutes within plate wells and washed three times in 1 x PBS. Within the cell culture plate wells, fixed cells were prepared for immunostaining by permeabilization, blocking, antibody incubations and washes. Antibody working dilutions were kept constant for all treatment conditions. Details on antibody manufacturer, catalog numbers, and working dilutions are described in detail elsewhere. [35] Automated immunofluorescence microscopy and mean fluorescent intensity measurements were performed with Biotek Cytation5 Cell Imaging Multimode Reader (Agilent, Santa Clara). Cell identity was calculated using thresholding parameters specific to each cell type for all DAPI^+^ cells. Resulting cell type proportions were calculated from cell thresholds using an in-house R script “CellFateQuant”. The source repository is maintained on GitHub at https://github.com/eraich26/CellFateQuant and is publicly available via Zenodo at DOI: https://doi.org/10.5281/zenodo.18644192. The code is released under the MIT license. All images were taken at 10X magnification unless otherwise noted.

### Study design and population

This study was approved by the Independent Review Board (IRB) at the Penn State College of Medicine (STUDY00013565). Written consent was obtained from all participants prior to enrollment. This study was conducted in accordance with the Declaration of Helsinki. Enrollment in this prospective, longitudinal cohort study occurred at a single academic institution from January 2020 – July 2024. Study team members identified opioid-exposed maternal/infant dyads through daily scanning of the newborn nursery charts and inpatient labor and delivery census lists. This screening was conducted by IRB-approved study personnel and was limited to the minimum necessary clinical information required to assess eligibility (gestational age and documented opioid exposure). If minimum requirements were met, a full chart review was conducted to confirm further eligibility. Additionally, nursing staff, neonatal/perinatal medicine fellows, and pediatric residents assisted in identification of eligible dyads. Identifiable health information accessed during the screening process was not recorded or retained unless full chart reviews were conducted to confirm eligibility prior to approaching for enrollment. All recorded patient health information was kept in a locked cabinet in a password- protected, locked laboratory. Mothers provided written informed consent for themselves and their infant to be enrolled in the study. Inclusion criteria for opioid-exposed maternal/infant dyads included 1) a gestational age > 35 weeks, 2) > 1 month of chronic *in utero* opioid exposure defined by chart documentation, maternal urine toxicology, and/or neonatal meconium toxicology results performed as part of routine clinical care and 3) delivery at Penn State Hershey Medical Center or transferred < 96 hours after birth. Exclusion criteria for opioid-exposed dyads included any of the following characteristics: < 35 weeks gestation, infant required mechanical ventilation or non-invasive mechanical support, infant exposed to magnesium sulfate, opioid-exposed infants who were actively receiving dextrose at the time of enrollment, major congenital anomalies, unable to provide consent, or dyads without a history of opioid exposure/dependence. Forty-six maternal infant dyads with chronic opioid exposure met minimal requirements were screened for study eligibility via full chart review. Thirty-six dyads met eligibility criteria and were approached for enrollment, and 28 consented for study participation. Of the 28 enrolled dyads, 16 infants did not receive pharmacotherapy. Twelve enrolled infants received pharmacotherapy. The primary clinical outcome was the maximum morphine dose (mg/kg) required for symptom control. The definition of the clinical outcome is further described below in ‘NOWS treatment protocol and severity measures’. Additionally, healthy maternal/infant dyad controls were enrolled in a separate study, as previously described. [52] Infant matching occurred at the individual level for gestational age, sex, and race. Mother/infant dyads enrolled in this separate study with history of maternal opioid use disorder or concurrent use of opioids were excluded for participant matching.

### Characterization of maternal and infant factors

The following medical and demographic measures were abstracted from both maternal and infant electronic medical records for all infants: infant sex, gestational age, race, and presence of *in utero* opioid exposure. For maternal/infant dyads with POE, the following measures were abstracted from both maternal and infant medical records: insurance type, birth weight, maternal age, maternal education, delivery type, feeding type, type of perinatal exposure, need for pharmacotherapy treatment, maximum morphine dose (mg/kg) required for symptom control, and the FNASS score at the time of sample collection. Access to medical records for research purposes began on January 15^th^, 2020. Protected patient health information, including identifiable information, was securely stored in an online RedCap database, in which only study personnel had access to during and after data collection. All written informed consent forms were securely stored in a locked cabinet in the study team’s locked research office.

### NOWS treatment protocol and severity measures

All opioid-exposed infants were observed for at least 48 hours in the newborn nursery rooming in with the infant’s mother, unless earlier pharmacologic therapy was indicated. At the mother’s discharge, infants were transferred to the neonatal intensive care unit (NICU) for three more days for observation of severe withdrawal symptoms requiring pharmacotherapy. The FNASS was used for all patients to measure withdrawal severity and to guide treatment initiation. During the time frame of which this study was conducted, the in-house protocol indicated pharmacotherapy treatment with three consecutive scores of eight or more. Participants with POE were further dichotomized into two subgroups depending on need for pharmacotherapy treatment of NOWS (yes/no). Maximal morphine dose required for symptom control was defined as the maximal morphine dose (mg/kg) that an infant required to maintain FNASS scores consistently below eight.

### Saliva collection, processing, and storage

Saliva samples were collected via buccal swabbing (DNA Genotek, Ottawa, Canada) at the time of enrollment, prior to initiation of morphine pharmacotherapy if indicated. Buccal swabs contained RNA stabilizer, allowing for immediate room temperature stabilization for up to 1 year. [53] To minimize contamination of saliva with feeds, swabs were only collected at least 30 minutes after the infant was last fed. Within 7 days of collection, saliva was aliquoted for later RNA extraction (250 μl) and a stock sample (remaining volume) and stored long term at −80°C. Saliva samples were labeled by participant ID. No patient identifiable information was labeled on saliva samples.

### Quantification of candidate miRNAs from saliva

Whole saliva RNA was extracted using the miRNeasy kit (Qiagen, Germantown), following the manufacturer’s protocol. RNA concentration was determined by benchtop spectrophotometry (Nanodrop, Fisher Scientific, Frederick). Polyadenylation and first strand synthesis was completed with 100 ng of RNA using MystiCq microRNA cDNA synthesis kit (Millipore Sigma, Burlington). Relative levels of target miRNA were analyzed by qPCR using MystiCq MicroRNA Assay primers, MystiCq MicroRNA Universal Reverse primers, and MystiCq MicroRNA SYBER Green MasterMix according to manufacturer’s instructions (Sigma, St. Louis). SNORD44 was used as an internal control. qPCR was completed on a CLIA-approved QuantStudio 5 machine (Applied Biosystems, Waltham). Non-templated controls were used for each primer. Melt curves were run for all reactions. Each sample was measured in technical triplicate. Delayed or absent amplification in any replicate was re-run. Missing continuous data was interpolated using group means.

### Statistics

For *in vitro* analyses, one-way ANOVAs were used to measure differences in transcript levels across cell conditions. A Mann-Whitney U test was used to measured transcript differences by transfection status (scramble vs. miRNA mimic). Ordinary two-way ANOVAs with Šídák’s multiple comparison test was used to assess effects of transfection status and treatment condition on cell identity proportions. Cell identity proportions in each treatment condition were not normalized to relative expression of transfected biotinylated miR-149-3p due to consistent transfection efficiencies between biological replicates. Historically, many overexpression studies transfect 100–300 nanomoles of single stranded miRNA mimics and assess transfection efficiency. At such high concentrations, we found that transfection efficiency was much wider than when transfecting 200 picomoles of dsRNA mimic (S1 Fig and S1 Table). Two-way ANOVAs with Tukey’s correction were used to analyze the interaction of cell fate identity with treatment condition and transfection status. All statistics were completed in Prism (GraphPad, San Diego).

For infant saliva related analyses, univariate comparisons for miRNA levels (i.e., infants with and without POE) were completed using Mann Whitney U tests. Further stratifications of these groups (i.e., miRNA levels in infants without POE vs. with POE not needing pharmacotherapy vs. and with POE needing pharmacotherapy) were compared using one-way ANOVAs. All continuous data had non-parametric distributions, as measured by D’Agnostino & Pearson, Anderson-Darling, Shapiro-Wilk, and Kolmogorov-Smirnov tests. Comparisons between maternal and infant sociodemographic factors were made using Mann Whitney tests for continuous variables or Fisher’s/Chi-square tests for categorical outcomes (Prism, GraphPad). Candidate miRNA levels measured in infant saliva at enrollment that were significantly different between treatment groups of infants with POE (i.e., pharmacologically treated vs. untreated) were added hierarchically into linear regression models for prediction of the maximum morphine dose required for infant symptom control (mg/kg). Accuracy, specificity, sensitivity and AUC values were reported. Akaike’s Information Criteria and adjusted R^2^ for each model was reported (Jamovi statistical software). [54]

## Results

### miR-149-3p levels are decreased in differentiating human iPSC-derived neural progenitors during chronic morphine exposure and further decreased in morphine withdrawal

**Fig 1:**
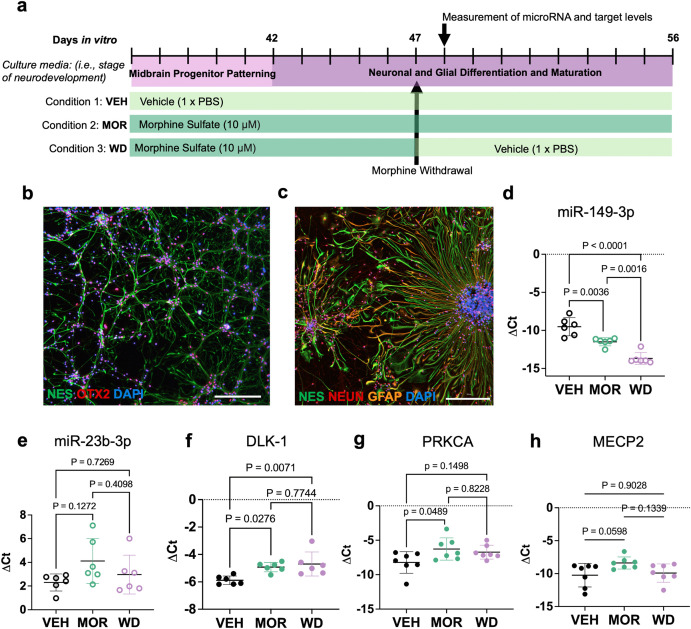
miR-149-3p levels are significantly decreased in human iPSC-derived midbrain neural progenitors during morphine exposure and morphine withdrawal. Treatment schematic during morphine exposure and withdrawal (a). hiPSC-derived midbrain neural progenitors are NES^+^ and OTX2^+^. (b). After differentiation on DIV56, there are NES^+^ neural progenitors, NEUN^+^ neurons, and GFAP^+^ astrocytes. (c). On DIV48, levels of miR-149-3p in differentiating neural progenitors were significantly decreased in morphine exposure and withdrawal conditions compared to vehicle-exposed controls (One way ANOVA, F = 34.18, *p* < 0.0001) (d). On DIV48, miR-23b-3p levels were unchanged during morphine exposure and withdrawal, 24 hours after washout (One way ANOVA, F = 2.218, *p* = 0.14) (e). On DIV48, putative transcript targets *DLK-1* (One way ANOVA, F = 7.27, *p* = 0.006) (f) and *PRKCA* (One way ANOVA, F = 3.59, *p* = 0.049) (g) were significantly increased in morphine exposure, and increased 24 hours after morphine withdrawal compared to vehicle (*p* = 0.007) (f,g). Additionally, there was no change in predicted target transcript *MECP2* between culture conditions (One way ANOVA, F = 3.45, *p* = 0.054) (h). All error bars represent mean + /- 1 SD. Scale bar represents 300 microns.

All cell treatment conditions are depicted in Fig 1a. As described previously, [35] hiPSC-derived midbrain neural progenitors are NES^+^ and OTX2^+^, indicating midbrain neural progenitor identity (Fig 1b). Upon completion of the differentiation protocol on DIV56, terminal cell fates include NEUN^+^ mature neurons, astrocytes that are GFAP^+^, or self-renewing NES^+^ progenitors (Fig 1c). To identify when to measure changes in miRNA levels after withdrawal, neural progenitors were harvested over a time course of 5, 12, 48, and 96 hours after morphine withdrawal stimulated by washout (S2 Fig). The most variation in miRNA levels between conditions occurred at 24 hours (S2a-e Fig). On DIV48, levels of miR-149-3p were significantly different between treatments (One way ANOVA, F = 34.18, *p* < 0.0001), decreased in morphine exposure relative to vehicle (Tukey’s multiple comparison test, *p* = 0.004), and further significantly decreased in morphine withdrawal relative to vehicle (Tukey’s, *p* < 0.0001) (Fig 1d). Levels of miR-23b-3p were not different between cell treatment conditions on DIV48 (One way ANOVA, F = 2.218, *p* = 0.14) (Fig 1e-f). Other opioid-responsive miRNAs with known roles in neurodevelopment, let-7a-5p and miR-192-5p, were not significantly different between treatment conditions 24 hours after morphine withdrawal (DIV48) (S2f and g Fig) (One way ANOVA, *p* > 0.05). Predicted transcript target of miR-149-3p *DLK1* [25] increased in morphine exposure and withdrawal, relative to vehicle controls (One way ANOVA, F = 7.28, *p* = 0.0062). All putative transcript levels were measured 24 hours after morphine washout. Another putative target of miR-149-3p, *PRKCA* [25], was elevated in morphine exposure (One way ANOVA, F = 3.596, *p* = 0.049) (Fig 1g), but its levels in morphine withdrawal were unchanged compared to those of vehicle-exposed neural progenitors (Tukey’s multiple comparisons, *p* = 0.15). There was no change in predicted target *MECP2* [25] across treatment conditions (One way ANOVA, F = 3.453, *p* = 0.0538) (Fig 1h). Levels of another predicted target, *SYNGAP1* [25]*,* was unchanged between culture conditions (S2h Fig).

### AGO2-enriched RNA immunoprecipitations confirm DLK1 as a target for miR-149-3p

**Fig 2:**
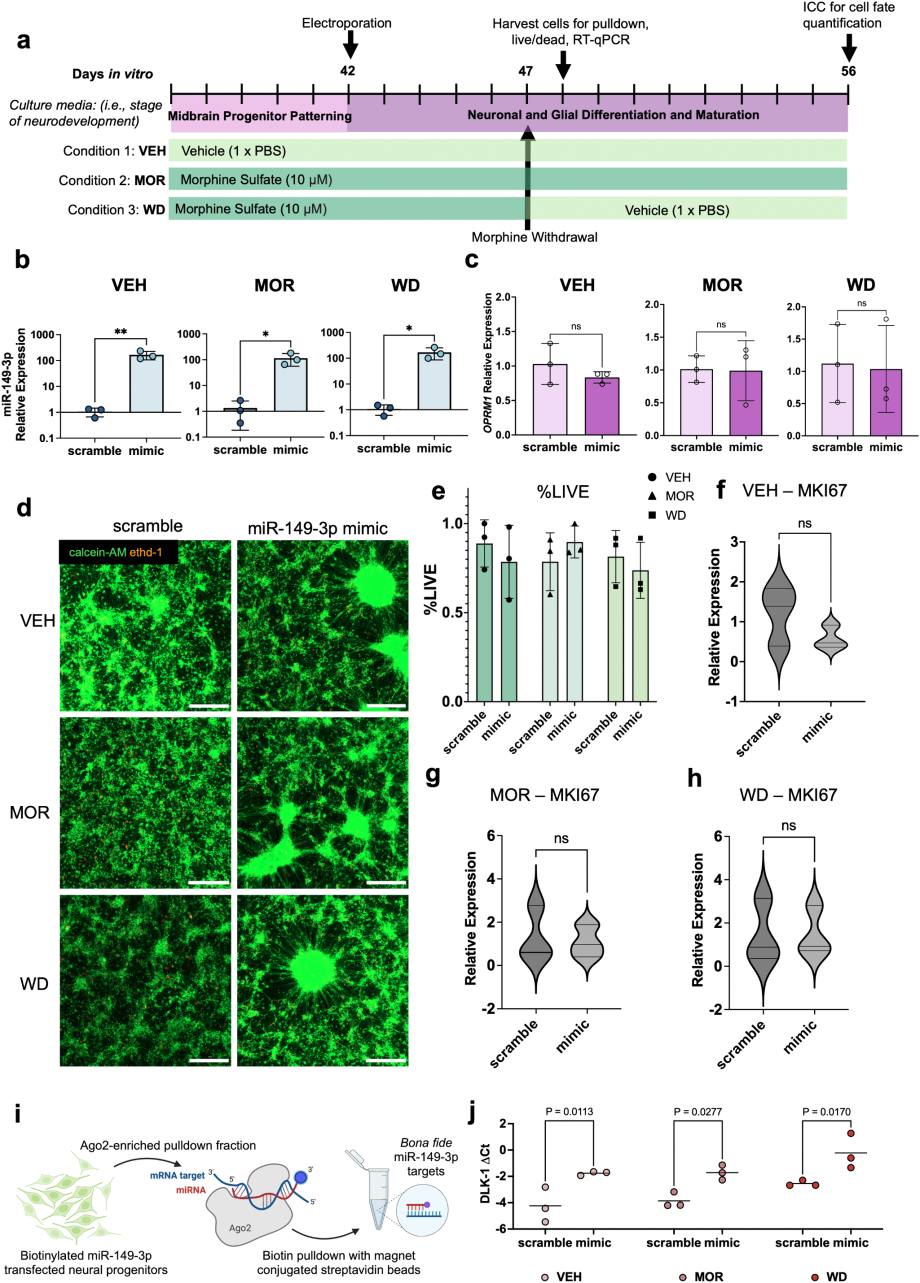
Transfection of miR-149-3p mimic binds to putative *DLK1* transcript while maintaining cell viability, opioid receptor and proliferation marker expression. Treatment schematic and experimental timeline **(a)**. Significantly increased expression of dsRNA biotinylated miR-149-3p was observed in mimic-transfected neural progenitors compared to dsRNA biotinylated cel-miR-243-3p scramble-transfected cells in vehicle (student t-test, two-tailed, p = 0.009), morphine and withdrawal treated conditions **(b)**. On DIV48, *OPRM1* transcript expression remained constant between mimic-transfected and scramble-transfected neural progenitors in vehicle (student t-test, two-tailed, p = 0.33), morphine-exposed (student t-test, two-tailed, p = 0.93), and withdrawal (student t-test, two-tailed, p = 0.88) treatment conditions **(c)**. LIVE/DEAD™ cytotoxicity staining for calcein-AM And EthD-1 was performed per manufacturer’s instructions and analyzed using the suggested microplate protocol in the manufacturer’s protocol **(d)**. There were no differences in proportions of alive cells between transfection status (Two-way ANOVA, F(1,12)= 0.10, p = 0.76) or treatment condition (Two way ANOVA, F(2,12) = 0.34, p = 0.72). There was no interaction between transfection status or treatment condition (F(2,12) = 0.87, p = 0.44) **(e)**. There were no changes to transcript levels of *MKI67* between transfection status in vehicle (student t-test, two-tailed, p = 0.24) **(f)**, morphine exposure (student t-test, two-tailed, p = 0.79) **(g)**, and withdrawal (student t-test, two-tailed, p = 0.98) treatment conditions **(h)**. Experimental schematic for AGO2-streptavidin double pulldown for immunoprecipitating miR-149-3p targets **(i)**. *DLK1* transcript had an increased relative expression in neural progenitor lysates transfected with biotinylated mimic compared to scramble (Two-way ANOVA, F(1,12) = 33.55, p < 0.001) in vehicle (Šidák’s multiple comparisons test, p = 0.011), morphine (Šidák’s multiple comparisons test, p = 0.023), and withdrawal (Šidák’s multiple comparisons test, p = 0.017) treatment conditions **(j)**.Three biological replicates were performed in triplicate for all assays. All error bars represent the mean + /- 1 SD. Scale bar represents 300 microns.

To confirm transcript targets of miR-149-3p, a double stranded biotinylated hsa-miR-149-3p mimic or scramble sequence cel-miR-243-3p was transfected into neural progenitors on DIV42 in three culture conditions (Fig 2a,b) prior to later RNA immunoprecipitations (DIV48). Despite the intentionally low stoichiometric ratio of miRNA mimic to cells, overexpression of miR-143-3p in samples transfected with mimic relative to scramble was noted across vehicle (student t-test, two tailed, p = 0.009), morphine exposed (student t-test, two-tailed, p = 0.028), and withdrawal conditions (student t-test, two tailed, p = 0.025) (Fig 2b) on DIV48. Transfection status did not affect levels of *OPRM1* transcript in vehicle (student t-test, two-tailed, p = 0.33), morphine exposure (student t-test, two-tailed, p = 0.93), or withdrawal treatment conditions on DIV48 (student t-test, two-tailed, p = 0.88) (Fig 2c). Transfection status also did not significantly affect the percent of alive cells on LIVE/DEAD™ cytotoxicity staining (Two-way ANOVA, F(1,12) = 0.10, p = 0.75), nor did treatment condition (Two-way ANOVA, F(2,12) = 0.34, p = 0.72) (Fig 2d,e). There was no interaction between transfection status and treatment condition (F(2, 12) = 0.87, p = 0.44). Additionally, transcript levels of marker of proliferation ki-67 (MKI67*)* were unchanged between mimic and scramble transfected neural progenitors across vehicle (student t-test, two-tailed, p = 0.25) (Fig 2f), morphine exposure (student t-test, two-tailed, p = 0.79) (Fig 2g) and withdrawal treatment conditions on DIV 48 (student t-test, two-tailed, p = 0.98) (Fig 2h). Neural progenitors were harvested 24 hours after morphine washout on DIV48 for AGO2 immunoprecipitation, followed by streptavidin purification. (Fig 2i). Increased relative expression of *DLK1* was present in mimic-transfected neural progenitors compared to scramble transfected cells across all treatment conditions (Fig 2j). Simple main effects analysis revealed that transfection status (i.e., scramble or mimic) had a statistically significant effect on relative *DLK1* levels in AGO2-enriched, streptavidin-pulldown lysates (Two way ANOVA, F (1, 12) = 33.55, p < 0.001). Additionally, simple main effects analysis showed that there was a statistically significant effect of treatment condition on relative *DLK1* levels (Two way ANOVA, F(2, 12) = 6.42, p = 0.013). There was not a significant interaction between effects of transfection status and treatment condition (Two-way ANOVA, F(2, 12) = 0.059, p = 0.94). Additionally, there was either inconsistent amplification of *PRKCA* or *MECP2* in RT-qPCR of AGO2-enriched pulldown samples (Supporting Fig S3). Together, these results show that transfection of a biotinylated double-stranded miRNA mimic at a low stoichiometric ratio of cells to mimic did not alter cell viability, *OPRM1* transcript receptor expression, or marker of proliferation *MKI67* transcript expression in any treatment condition. Further, double pulldown immunoprecipitations of AGO2 and streptavidin revealed an increase in bound *DLK1* transcript in mimic-transfected cells relative to scramble-transfected cells in each treatment condition.

### Overexpression of miR-149-3p during chronic morphine exposure attenuates alteration of NEUN^+^ neuron proportions in a DLK1-independent manner

**Fig 3:**
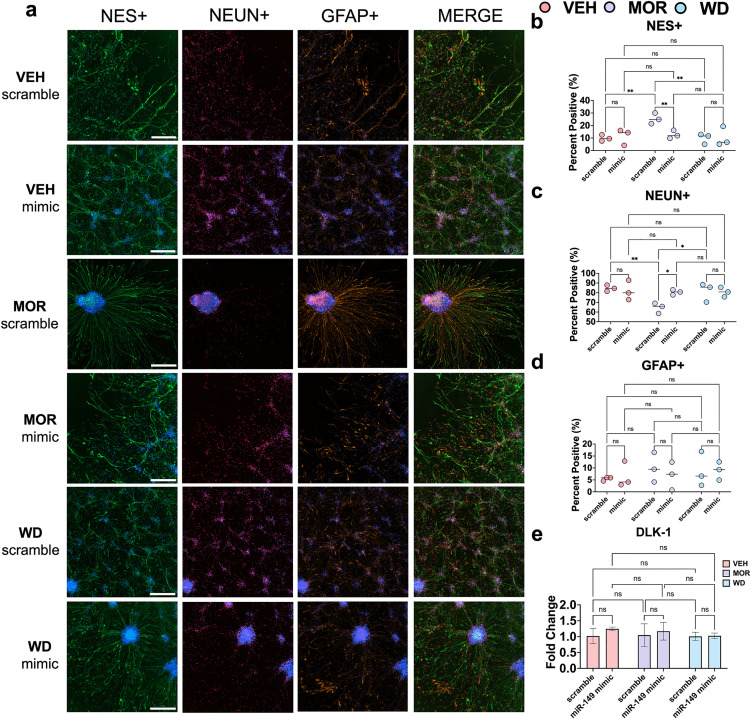
miR-149-3p associated changes in cell fate proportions are DLK-1 independent. Immunostaining was completed on DIV56 for NES^+^ (green), GFAP^+^ (orange), and NEUN^+^ (red) cell identities. DAPI stain (blue) was used as a primary mask to identify the total number of cells in an image. Quantitative microscopy using previously established thresholding parameters was performed using Cytation5 (Biotek). Scale bar represents 300 microns (a). Proportions of NES+ cells were compared across transfection status and treatment condition. There was a statistically significant decrease in NES^+^ cell proportions during chronic morphine exposure in cells that were transfected with miR-149 mimic compared to scramble (Two-way ANOVA, Šidák’s multiple comparisons test, 25.46% vs. 12.62% MOR scramble vs. MOR mimic, adj. p = 0.009) (b). Transfection of miR-149-3p mimic attenuated alteration of NEUN+ cell proportions in cells that were exposed to chronic morphine exposure compared to scramble controls (Two-way ANOVA, Šidák’s multiple comparisons test, 64.5% vs. 80.5% MOR scramble vs. WD scramble, adj. p = 0.013) (a,c). No significant changes in resulting GFAP^+^ proportions were observed due to transfection status in any condition (Two-way ANOVA, F(1,12) = 0.058, p = 0.81) (d). Relative expression of DLK-1 transcript was unaffected by transfection status (Two-way ANOVA, F(1,12) = 1.37, p = 0.26) or treatment condition (Two-way ANOVA, F(2,12) = 0.48, p = 0.63 (e).

We previously showed in non-electroporated hiPSC-derived neural progenitors that chronic morphine exposure decreased the proportions of resulting NEUN^+^ neurons in favor of NES^+^ neural progenitors, and that these proportions normalized with morphine withdrawal, simulated by washout [35]. To explore the role of miR-149-3p and its binding target *DLK1* in resulting cell fate proportions, neural progenitors were transfected with either a miR-149-3p mimic or scramble cel-miR-243-3p sequence via electroporation on DIV42, and differentiated in three treatment conditions as described above (Fig. 2a). Differentiated cell types were fixed and immunostained on DIV56 for cell identity markers NES, GFAP, and NEUN (Fig. 3a). We found that scramble-transfected neural progenitor cells differentiated to similar resulting proportions of cell fates as described previously. [35] For example, in scramble-transfected cells, chronic morphine exposure significantly increased proportions of NES^+^ neural progenitors relative to vehicle controls (Two-way ANOVA, Šidák’s multiple comparisons test, mean proportions 9.9% vs. 25.5%, VEH vs. MOR, adj. p = 0.008) (Fig 3b). Scramble-transfected neural progenitors that experienced withdrawal after chronic morphine exposure displayed NES^+^ cell proportions similar to those of vehicle controls (Two-way ANOVA, Šidák’s multiple comparisons test, mean proportions 25.5% vs. 9.8%, MOR vs. WD, p = 0.008) (Fig 3b). Transfection of miR-149-3p mimic had no effect on NES^+^ proportions in vehicle exposed neural progenitors compared to scramble controls (Two-way ANOVA, Šidák’s multiple comparisons test, mean proportions 9.9% vs. 11.5%, VEH scramble vs. VEH mimic, adj. p = 0.72) (Fig. 3b). In chronic morphine-exposed neural progenitors, proportions of NES^+^ cells were significantly decreased in the group that was transfected with miR-149-3p mimic compared to scramble (Two-way ANOVA, Šidák’s multiple comparisons test, mean proportions 25.5% vs. 12.62%, MOR scramble vs. MOR mimic, adj. p = 0.009), and returned to vehicle exposed levels (Two-way ANOVA, Šidák’s multiple comparisons test, mean proportions 11.5% vs. 12.6%, VEH mimic vs. MOR mimic, adj. p = 0.99) (Fig 3b). There was no effect of miR-149-3p mimic transfection on NES^+^ cell proportions in the morphine withdrawal condition (Two-way ANOVA, Šidák’s multiple comparisons test, 9.8% vs. 10.4% WD scramble vs. WD mimic, adj. p = 0.99) (Fig 3b). In summary, overexpression of miR-149-3p in neural progenitors that were differentiated toward terminal cell fates in the presence of chronic morphine attenuated alteration of NES^+^ cell type proportions relative to non-transfected controls.

Similarly to NEUN^+^ cell proportion trends we previously reported, [35] we observed that proportions of scramble-transfected NEUN^+^ cells during chronic morphine exposure were significantly decreased compared to levels of scramble-transfected cells in vehicle exposure (Two-way ANOVA, Šidák’s multiple comparisons test, 84.6% vs. 64.5% VEH scramble vs. MOR scramble, adj. p = 0.01) (Fig 3c). As observed in vehicle-exposed NES^+^ cells (Fig 2b), there was no effect of miR-149-3p overexpression compared to scramble-transfected controls (Two-way ANOVA, Šidák’s multiple comparisons test, 84.6% vs. 81.89% VEH scramble vs. VEH mimic, adj. p = 0.63) (Fig 3c). Overexpression of miR-149-3p in cells chronically exposed to morphine significantly increased proportions of NEUN^+^ cells (Two-way ANOVA, Šidák’s multiple comparisons test, 64.51% vs. 80.52% MOR scramble vs. MOR mimic, adj. p = 0.013) (Fig 3c), rescuing NEUN^+^ proportions to those of vehicle-exposed levels (Two-way ANOVA, Šidák’s multiple comparisons test, 81.89% vs. 80.52% VEH mimic vs. MOR mimic, adj. p = 0.99) (Fig 3c). Mirroring NEUN^+^ cell proportion trends we previously reported, morphine withdrawal significantly increased NEUN^+^ proportions in scramble-transfected cells compared to those in the morphine-exposed condition (Two-way ANOVA, Šidák’s multiple comparisons test, 64.51% vs. 81.45% MOR scramble vs. WD scramble, adj. p = 0.0.027) (Fig 3c). However, overexpression of miR-149-3p had no effect on NEUN^+^ proportions compared to withdrawal scramble controls (Two-way ANOVA, Šidák’s multiple comparisons test, 81.45% vs. 80.71% WD scramble vs. WD mimic, adj. p = 0.90) (Fig 3c). Neither transfection status (Two-way ANOVA, F(1,12) = 0.058, p = 0.81) nor treatment condition (Two-way ANOVA, F(2,12) = 0.47, p = 0.63) affected resulting GFAP^+^ cell proportions. Notably, there were no differences in resulting GFAP^+^ cell fate proportions in scramble-transfected cells between morphine and withdrawal exposure treatments (Two-way ANOVA, Šidák’s multiple comparisons test, 10.0% vs. 8.73% MOR scramble vs. WD scramble, adj. p = 0.99) (Fig 3d). In summary, miR-149-3p overexpression attenuated the alteration of NEUN^+^ cell type proportions after neural differentiation in the presence of chronic morphine exposure relative to non-transfected controls.

To assess if levels of *DLK1* transcript from cell total RNA were altered in response to overexpression of miR-149-3p levels, differentiating hiPSC-derived neural progenitors were harvested on DIV48 for total RNA extraction and processed for RT-qPCR (Fig 2a). Neither transfection status (Two-way ANOVA, F(1, 12) = 1.37, p = 0.26) nor treatment condition (Two-way ANOVA, F(2, 12) = 0.48, p = 0.63) influenced levels of DLK1 transcript levels (Fig 3e).

### Participant cohort of infants with POE

**Fig 4:**
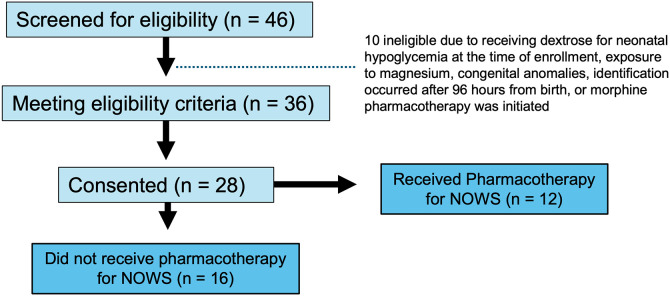
CONSORT Diagram for participants with POE. Schematic for the number of participants who were screened (46), eligible (36), consented (28), received pharmacotherapy (12), did not receive pharmacotherapy (16).

Eligibility screening details for infants with POE are shown in the CONSORT diagram in Fig 4. Most participants were female (29/56, 51.8%), White race (43/56, 76.8%), and non-Hispanic (52/56, 92.9%) (Table 1). There were no differences in infant race, sex, and gestational age or maternal race between groups of infants exposed and unexposed to POE (Table 1). Mothers of infants with POE were less likely to have a college degree (7.1% vs. 53.6%, p < 0.0001) and more likely to be covered by Medicaid than by other types of health insurance (85.7% vs. 17.9%, p < 0.0001) (Table 1) than mothers of unexposed infants. Next, infants with POE were stratified by need for treatment (Table 2). Infants that required treatment (n = 12), compared to those that were exposed but did not require treatment, were more likely to be female (odds ratio (OR), 0.09; 95% CI, 0.017 to 0.54, p = 0.009). Mothers with infants with POE that required treatment were more likely to be older (95% CI [0.0,7], p = 0.04). There were no significant differences in infant birth weight, feeding type, neonatal complications, or FNASS scoring at the time of sample collection between infants that did and did not require pharmacotherapy for NOWS symptoms. Additionally, there were no differences in additional tobacco or polysubstance exposures between groups. Notably, there were no differences in FNASS scores at the time of sample collection between those that did and did not require morphine pharmacotherapy (5.67 vs. 4.31, p = 0.25) (Table 2). Finally, there were no differences in maternal education level (p = 0.67) or insurance type (p = 0.74) between infants that did and did not require morphine pharmacotherapy (Table 2).

**Table 1:** Maternal and infant demographic information for infants with and without POE. α = 0.05. ^†^Fisher’s Exact Test, ^#^Mann Whitney Test (two-tailed), ^‡^Chi-square.

	Infants with POE (N = 28)	Infants without POE (N = 28)	Overall (N = 56)	p value
**Sex**				>0.99^†^
Male	13 (46.4%)	14 (50.0%)	27 (48.2%)	
Female	15 (53.6%)	14 (50.0%)	29 (51.8%)	
**Gestational Age at Birth**				0.53^#^
Mean (SD)	38.9 (0.885)	38.9 (0.962)	38.9 (0.917)	
Median [Min, Max]	39.2 [37.0, 40.1]	39.1 [37.2, 40.4]	39.1 [37.0, 40.4]	
**Infant Race**				0.63^†^
Asian	0 (0%)	0 (0%)	0 (0%)	
Bi-racial	3 (10.7%)	1 (3.6%)	4 (7.1%)	
Black	2 (7.1%)	4 (14.3%)	6 (10.7%)	
White	21 (75.0%)	22 (78.6%)	43 (76.8%)	
Other	2 (7.1%)	1 (3.6%)	3 (5.4%)	
**Infant Hispanic?**				0.61^†^
No	25 (89.3%)	27 (96.4%)	52 (92.9%)	
Yes	3 (10.7%)	1 (3.6%)	4 (7.1%)	
**Maternal Race**				0.75^†^
Asian	0 (0%)	1 (3.6%)	1 (1.8%)	
Bi-racial	1 (3.6%)	0 (0%)	1 (1.8%)	
Black	2 (7.1%)	4 (14.3%)	6 (10.7%)	
White	23 (82.1%)	22 (78.6%)	45 (80.4%)	
Other	2 (7.1%)	1 (3.6%)	3 (5.4%)	
**Type of Exposure**				–
Buprenorphine	6 (21.4%)	0 (0%)	6 (10.7%)	
Buprenorphine/Naloxone	7 (25.0%)	0 (0%)	7 (12.5%)	
Heroin	1 (3.6%)	0 (0%)	1 (1.8%)	
Hydrocodone	1 (3.6%)	0 (0%)	1 (1.8%)	
Hydromorphone	1 (3.6%)	0 (0%)	1 (1.8%)	
Methadone	9 (32.1%)	0 (0%)	9 (16.1%)	
Morphine	1 (3.6%)	0 (0%)	1 (1.8%)	
Oxycodone	2 (7.1%)	0 (0%)	2 (3.6%)	
None	0 (0%)	28 (100%)	28 (50.0%)	
**Educational Level**				<0.0001^‡^
Some high school	4 (14.3%)	0 (0%)	4 (7.1%)	
High school graduate	8 (28.6%)	1 (3.6%)	9 (16.1%)	
Some college	12 (42.9%)	6 (21.4%)	18 (32.1%)	
Completed College	2 (7.1%)	15 (53.6%)	17 (30.4%)	
Post graduate degree	0 (0%)	6 (21.4%)	6 (10.7%)	
Unknown	2 (7.1%)	0 (0%)	2 (3.6%)	
**Health Insurance Type**				<0.0001^†^
Medicaid	24 (85.7%)	5 (17.9%)	29 (51.8%)	
Uninsured	2 (7.1%)	0 (0%)	2 (3.6%)	
Private	2 (7.1%)	22 (78.6%)	24 (42.9%)	
Military	0 (0%)	1 (3.6%)	1 (1.8%)	

**Table 2:** Infant medical and sociodemographic data between infants treated and untreated for NOWS. α = 0.05. ^†^Fisher’s Exact Test, ^#^Mann Whitney Test (two-tailed), ^‡^Chi-square.

	Opioid-Exposed, Not Treated (N = 16)	Opioid-Exposed, Treated (N = 12)	Overall (N = 28)	p value
**Sex**				0.009^†^
Female	5 (31.3%)	10 (83.3%)	15 (53.6%)	
Male	11 (68.8%)	2 (16.7%)	13 (46.4%)	
**Gestational Age**				0.12^#^
Mean (SD)	39.1 (0.878)	38.7 (0.869)	38.9 (0.885)	
Median [Min, Max]	39.3 [37.1, 40.1]	39.1 [37.0, 39.5]	39.2 [37.0, 40.1]	
**Race**				0.12^†^
White	14 (87.5%)	7 (58.3%)	21 (75.0%)	
Black	1 (6.3%)	1 (8.3%)	2 (7.1%)	
Bi-racial	0 (0%)	3 (25.0%)	3 (10.7%)	
Other	1 (6.3%)	1 (8.3%)	2 (7.1%)	>0.99^†^
**Hispanic**				
Yes	2 (12.5%)	1 (8.3%)	3 (10.7%)	
No	14 (87.5%)	11 (91.7%)	25 (89.3%)	
**Birth Weight**				0.35^#^
Mean (SD)	3210 (475)	3010 (446)	3120 (465)	
Median [Min, Max]	3140 [2480, 4180]	3010 [2220, 3620]	3080 [2220, 4180]	
**Delivery Type**				>0.99^†^
Cesarean	10 (62.5%)	8 (66.7%)	18 (64.3%)	
Vaginal	6 (37.5%)	4 (33.3%)	10 (35.7%)	
**APGAR at 1 min**				0.13^#^
Mean (SD)	8.13 (0.500)	7.25 (1.71)	7.75 (1.24)	
Median [Min, Max]	8.00 [7.00, 9.00]	7.50 [4.00, 9.00]	8.00 [4.00, 9.00]	
**APGAR at 5 min**				0.44^#^
Mean (SD)	8.88 (0.342)	8.50 (1.00)	8.71 (0.713)	
Median [Min, Max]	9.00 [8.00, 9.00]	9.00 [6.00, 9.00]	9.00 [6.00, 9.00]	
**Complications**				>0.99^†^
Hypoglycemia	1 (6.3%)	0 (0%)	1 (3.6%)	
Possible fetal heart complication	1 (6.3%)	0 (0%)	1 (3.6%)	
Respiratory distress	2 (12.5%)	2 (16.7%)	4 (14.3%)	
None	12 (75.0%)	10 (83.3%)	22 (78.6%)	
**Prenatal Exposure**				0.57^†^
Buprenorphine	5 (31.3%)	1 (8.3%)	6 (21.4%)	
Buprenorphine/Naloxone	3 (18.8%)	4 (33.3%)	7 (25.0%)	
Heroin	1 (6.3%)	0 (0%)	1 (3.6%)	
Hydrocodone	1 (6.3%)	0 (0%)	1 (3.6%)	
Hydromorphone	1 (6.3%)	0 (0%)	1 (3.6%)	
Methadone	4 (25.0%)	5 (41.7%)	9 (32.1%)	
Oxycodone	1 (6.3%)	1 (8.3%)	2 (7.1%)	
Morphine	0 (0%)	1 (8.3%)	1 (3.6%)	
**Feeding Type**				0.83^†^
Both	11 (68.8%)	10 (83.3%)	21 (75.0%)	
Breast	2 (12.5%)	1 (8.3%)	3 (10.7%)	
Formula	3 (18.8%)	1 (8.3%)	4 (14.3%)	
**Average Finnegan Score at Saliva Collection**				0.25^#^
Mean (SD)	4.31 (3.36)	5.67 (3.03)	4.89 (3.24)	
Median [Min, Max]	3.50 [0, 11.0]	5.50 [2.00, 12.0]	5.00 [0, 12.0]	
**Maximum Morphine Dose (mg/kg)**				<0.0001^#^
Mean (SD)	0 (0)	0.0706 (0.0252)	0.0302 (0.0390)	
Median [Min, Max]	0 [0, 0]	0.0656 [0.0399, 0.116]	0 [0, 0.116]	
**Maternal Age**				0.04^#^
Mean (SD)	29.1 (4.60)	32.4 (4.17)	30.5 (4.65)	
Median [Min, Max]	27.5 [23.0, 37.0]	32.0 [27.0, 38.0]	30.5 [23.0, 38.0]	
**Tobacco Use**				0.27^†^
Current user	8 (50.0%)	3 (25.0%)	11 (39.3%)	
Former user	0 (0%)	1 (8.3%)	1 (3.6%)	
Never user	7 (43.8%)	8 (66.7%)	15 (53.6%)	
Household tobacco exposure	1 (6.3%)	0 (0%)	1 (3.6%)	
**Marijuana use**				>0.99^†^
No	13 (81.3%)	9 (75.0%)	22 (78.6%)	
Yes	3 (18.8%)	3 (25.0%)	6 (21.4%)	
**Other polysubstance use**				0.56^†^
No	15 (93.8%)	10 (83.3%)	25 (89.3%)	
Yes	1 (6.3%)	2 (16.7%)	3 (10.7%)	
**Maternal Education**				0.67^†^
High school or less	12 (75%)	10 (83.3%)	22 (78.6%)	
Some college or completed college	4 (25%)	2 (16.67%)	6 (21.4%)	
**Insurance Type**				
Medicaid	13 (81.2%)	11 (91.7%)	24 (85.8%)	0.74^†^
Uninsured	1 (6.3%)	1 (8.3%)	2 (7.1%)	
Private	2 (12.5%)	0 (0%)	2 (7.1%)	

### Saliva miR-149-3p levels predicted maximum morphine dose required for symptom control

**Fig 5:**
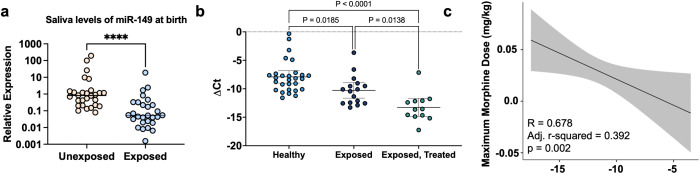
Saliva miR-149-3p levels predict maximum morphine dose required for infant symptom control. Levels of miR-149-3p were significantly decreased in saliva of infants with POE compared to unexposed infant controls (Mann Whitney U test, p < 0.0001) **(a)**. Levels of miR-149-3p were significantly decreased in infants treated for NOWS compared to infants only with POE (One-way ANOVA, F = 17.24, p < 0.0001) **(b)**. Adding salivary levels of miR-149-3p hierarchically into a model using infant sex and FNASS as predictors improved the prediction model **(c)**.

Levels of salivary miR-149-3p were lower in infants with POE compared to unexposed controls at enrollment (between birth and 96 hours of life) (95% CI [−1.05, −0.18], p < 0.0001) (Fig 5a). When stratifying infants with POE by receipt of NOWS pharmacotherapy, levels were significantly lower in those that were treated compared to those that were not (One-way ANOVA, F = 17.24, Tukey’s multiple comparisons test, p = 0.014) (Fig 5b). Out of the reported factors in Table 2, only infant sex (β = −0.030, SE = 0.013, 95% CI [−0.056, 0.003], p = 0.03), and FNASS (β = 0.004, SE = 0.0018, 95% CI [1.28 x 10^-4^, 0.008], p = 0.043) displayed a relationship with maximum morphine dose on an omnibus ANOVA test. Using hierarchical linear regression, a model controlling for factors of infant sex and mean FNASS at the time of saliva collection as a covariate accounted for 29% of the variance in maximum morphine dose required for infant symptom control (mg/kg) (R = 0.583, adj. R^2^ = 0.29, AIC = −106, F = 6.42, p = 0.006). Adding levels of miR-149-3p (β = −0.0052, SE = 0.002, 95% CI [−0.10, −5.55 x 10^-4^], p = 0.03) to the model improved the overall performance (R = 0.678, adj. R^2^ = 0.392, AIC = −110, F = 6.81, p = 0.002). Approximately, a 5 unit drop in the level of miR-149-3p is associated with an approximate 0.025 mg/kg increase in morphine dose required for infant symptom control (Fig 5c).

As an additional model, we sought to explore if maternal education level or insurance type impacted salivary miR-149 levels in infants with POE. Using hierarchical linear regression, a model controlling for the factor of maximum morphine treatment dose (β = −36.5, SE = 12.58, 95% CI [−62.3, −10.59], p = 0.008) accounted for 21.5% of the variance in miR-149 level (R = 0.494, adj. R^2^ = 0.21, AIC = 137, F = 8.40, p = 0.008). Adding in factors of maternal education level and insurance type to the model decreased the overall performance (R = 0.513, adj. R^2^ = 0.263, AIC = 142, F = 2.05, p = 0.120).

## Discussion

The goal of this study was two-fold: 1) to use a hypothesis-driven approach for characterizing the physiologic role of opioid-responsive miRNAs in neural stem cell fate commitments, and 2) to assess if miRNAs identified *in vitro* possessed clinical utility for infants with POE. A previous literature search identified miRNAs miR-149 [55], miR-192 [36,56,57], let-7a[38,39], and miR-23b[48] as miRNAs with either experimentally or predicted regulatory roles at the intersection of these processes. Here, we showed that miR-149-3p was the only miRNA candidate altered in human iPSC-derived midbrain neural progenitors during chronic morphine exposure. Using miRDB, we identified putative mRNA targets of miR-149 that are implicated in neurodevelopment and opioid signaling, (*DLK1*, *MECP2*, *PRKCA*, *SYNGAP1*) and identified two transcripts that responded to morphine exposure, *DLK1* and *PRKCA*. We sought to experimentally confirm miRNA:mRNA binding with a dsRNA biotinylated mimic of miR-149. Only *DLK1* transcripts had increased levels in AGO2-enriched double pulldown immunoprecipitations compared to scramble-transfected controls. Despite a confirmed interaction between miR-149-3p and *DLK1* transcript using AGO2-enriched RNA immunoprecipitation methods, cell lysate total RNA levels of *DLK1* were unchanged with transfection of miR-149-3p mimic. This may mean that miR-149-3p can bind to *DLK1*, but the interaction may not result in mRNA degradation under these experimental conditions. Therefore, it appears that overexpression of miR-149-3p improved proportions of resulting NEUN^+^ neurons and reset proportions of NES^+^ progenitors during chronic morphine exposure, in a DLK1-independent manner.

Given what is known about miR-149-3p, its role in neural stem cell fate commitments and opioid signaling may not be surprising. miR-149-3p is broadly expressed throughout the brain in development. [58] One study previously noted that miR-149-3p was responsive to neurotoxic stimuli in hiPSC-derived neural stem cells, and a regulator of neural differentiation. [23] It appears that miR-149-3p has a neuroprotective role; there is evidence that single nucleotide polymorphisms in its pre-miRNA sequence are associated with increased stroke risk [59] and that levels of miR-149-3p are significantly decreased in Alzheimer’s Disease (AD) patient serum compared to controls. [42] Further, decreasing serum levels of miR-149-3p predicted worsening cognitive outcomes of AD patients. [42] Additionally, miR-149-3p is a known regulator of toll-like receptor 4 (TLR4). TLR4 recognizes pathogen-associated molecular patterns (PAMPs) and danger-associated molecular patterns (DAMPs), and binds opioids in a manner similar to lipopolysaccharide (LPS). [60] Interestingly, TLR4 has low-affinity for morphine, fentanyl, and oxycodone by binding to the LPS-binding pocket of myeloid differentiation-2 (MD-2). [28] Opioid binding subsequently stimulated nuclear factor kappa-light-chain-enhancer (NFκB) for release of pro-inflammatory cytokines. [28] In preclinical models of prenatal methadone exposure, methadone increased expression of cerebral *TLR4* and myeloid differentiation primary response 88 (Myd88*)* mRNAs, microglial activation, and pro-inflammatory cytokines in exposed pups compared to saline controls. These observations led to cognitive deficits and brain injury in exposed rat pups compared to controls. [61] Other known targets of miR-149-3p include cell division cycle 42 (CDC42) and B-cell lymphoma (BCL2). [62] While we did not observe differences in cellular proliferation between transfection status or treatment condition by measuring levels of *MKI67* transcript, future studies may want to explore overexpression of miR-149-3p on other cell-cycle related transcripts in neural stem cells.

DLK1 is a known negative regulator of differentiation, as it directly inhibits NOTCH1 to maintain the balance between neural stem cell renewal and differentiation. [63,64] Deletion of *DLK1* in neural stem cells resulted in differentiation of mature neurons and subsequent loss of progenitors. [65] In non-neuroepithelial cells, *DLK1* overexpression delays differentiation and increases precursor proliferation. [66] Despite well-documented roles of DLK1 in regulating neural stem cell differentiation and it being a target of miR-149-3p, we did not observe miR-149-3p-mediated differences in *DLK1* levels by transfection status or treatment condition. There are a few potential reasons that we did not observe differences in *DLK1* levels (derived from total cell RNA, not AGO2 enriched fractions) with miR-149-3p transfection during opioid exposure. First, we chose to do AGO2-enriched biotin pulldowns to confirm miR-149-3p binding to *DLK1*. Transfection with miR-149 mimic was sufficient to increase bound *DLK1* in the AGO2 complex, but not sufficient to decrease bulk cellular levels of *DLK1*. It is possible that an increased concentration of miR-149 mimic could significantly impact bulk *DLK1* levels, but our preliminary results suggest such a procedure would also impact transfection efficiency variation, confounding our ability to interpret the findings (S1 Fig). We focused on measuring the levels of *DLK1* at the transcript level, as our study was focused on the mechanism of action of miRNAs, though future studies interested in downstream mediators or protein-protein interactions could measure DLK1 protein levels in co-cultures. miRNAs have been shown to achieve target knockdown within 24 hours of transfection [67,68], which is why we were mainly interested in measuring DLK1 levels 24 hours after miR-149 mimic transfection. In future studies, more time points throughout differentiation are likely needed to detect more nuanced changes in *DLK1* and other targets after differentiation. Additionally, miR-149-3p mediated changes in *DLK-1* secondary to chronic morphine exposure may be cell-type specific. For example, only NEUN^+^ and NES^+^ cell types exhibited differences in resulting proportions, whereas GFAP^+^ astrocytes did not. Bulk RNA was isolated from all cells at DIV48, which may have clouded heterogeneity within early cell identities. It has been previously noted that GFAP^+^ astrocytes differentially process DLK1 protein than neural stem cells and neurons. [65]

Though these results suggest that miR-149-3p overexpression attenuates differences in cell type proportions following opioid exposure, it is not dependent upon reductions in *DLK1*. Thus, other transcriptional targets of miR-149-3p should be explored. Future studies may want to bulk RNA sequence AGO2-enriched biotin-pulldown RNAs for identifying strong mRNA target candidates. Other limitations of the study included use of only one line. We used a healthy female donor control line with 78.2% European and 21.8% South Asian ancestry [44]. While there have been calls for inclusion of more female and ethnically diverse iPSC lines in models of POE [69], our results may not be generalizable to other lines that differ by genetic background and sex. Our rationale for using only one cell line was rooted in the proof-of-concept nature of our culture model, as previously described [35]. Briefly, we established that a longer length of differentiation from iPSC to monolayer derived neural progenitor (~50 days *in vitro*) was required for optimal expression of mu and kappa opioid receptors in neural progenitors using SCTi003-a. Available resources were limited for reproducibility in other lines using the same differentiation protocol length.

Previous studies have highlighted sex-specific differences in neuronal microRNA expression, namely in animal-derived tissues, which have circulating sex steroids in the brain. In human-derived cultures, 17β-estradiol can be produced by neurons from added androgen precursors, which are converted through the endogenously expressed aromatase enzyme [70]. While we did not add any exogenous androgen precursors to culture media, it’s possible that some iPSC-derived cells were capable of steroidogenic production. It has been previously shown that the presence of estradiol influences microRNA response, especially in the presence of a stressor. [71] The “stressor” in the case of our cultures was morphine. If the response to morphine was affected due to presence of estrogen, our microRNA results may be female-specific. This important limitation could have affected not only microRNA expression, but also differences in the proportions of differentiated iPSC-derived neural cells. Of note, miR-149 is not one of the 118 X-linked microRNAs, of which could escape X-chromosome inactivation and have higher expression in females [72]. Also, we did see reproducibility of *in vitro* miR-149 trends in our cohort of female and male infant saliva samples.

Other epigenetic mechanisms worth further investigating in future human stem cell studies is the role of sex-specific differences on imprinted genes. It is well known that *DLK1* is maternally imprinted and paternally expressed. There is evidence to suggest that imprinting patterns may differ between sexes. For example, one study showed that levels of imprinted genes were higher in males than females. [73] Another study profiled the expression of miRNAs and protein-encoding genes at the DLK1-DIO3 locus in males and female patients with relapsing-remitting multiple sclerosis. While there were a cluster of miRNAs at this locus that were upregulated in males but not female patients, there were no differences in DLK1 expression in males or females. [74] Nevertheless, how somatic reprogramming of in iPSC lines may affect *DLK1* expression is unknown, and we did not evaluate imprinting status in this female-donor derived line. Further, our reliance on iPSCs from a female donor prevents us from assessing whether similar sex-specific dynamics would be observed in male iPSCs. Future studies should validate findings in more than one iPSC donor line, in both male and female derived lines.

We also employed a translational aspect to our study to further to probe if opioid-responsive miRNAs identified *in vitro* would have clinical utility for infants with POE. Using a hypothesis-driven approach using a cohort of 56 infants, we identified that miR-149-3p was significantly decreased in infants with POE compared to unexposed infant controls. Additionally, miR-149-3p further decreased in infants that later required pharmacologic therapy for NOWS (i.e., experienced more severe withdrawal symptoms). Thus, the lowest levels of miR-149-3p in infant saliva at birth were seen in infants that would go on to develop NOWS and require treatment. A hierarchical linear regression model utilizing levels of miR-149-3p increased ability to predict the maximum morphine dose needed for symptom control, compared to using infant clinical factors alone. We showed that approximately a 5 unit drop in the level of miR-149-3p (ΔCt) correlated with a 0.025 mg/kg dose increase in morphine for infant symptom control. Given that the morphine dose for initiation of NOWS pharmacotherapy is 0.05 mg/kg [75], this may be a clinically meaningful finding. We want to emphasize that these results are preliminary, based on a small cohort of 28 infants. Our results should be interpreted with careful consideration of model stability and residual confounding and could be considered as exploratory. Future studies should investigate the utility of miRNAs in infant saliva in a larger cohort of opioid-exposed infants. A future goal would be to identify a biomarker-informed morphine dose for infants with POE that could minimize total opioid exposure during hospital treatment for withdrawal symptoms, compared to following a standard titration system that many hospitals employ. Also, such a biomarker-informed morphine dose could reduce overall time in the hospital. A saliva-based miRNA biomarker test would be non-invasive, cheap, accessible, and quick to process as benchtop qPCR machines are standard in clinical laboratories.

Several maternal and infant sociodemographic factors were profiled for 1) unexposed vs. infants with POE and 2) infants with POE that did and did not require pharmacotherapy. For infants who required pharmacotherapy for NOWS symptoms, maternal age was increased compared to those of infants that did not. This is likely an artifact secondary to the small sample size, as maternal age has not shown a relationship to the need for pharmacotherapy in larger studies. [39] Because maternal age was not a significant predictor of maximal morphine dose, it was not included as a covariate in this study’s prediction models. Additionally, substantial literature has linked various socioeconomic factors such as degree of poverty and maternal education to infant withdrawal severity [76,77] and neurodevelopmental outcomes [78,79]. In our cohort of infants with POE, insurance type and maternal education level did not differ by need for withdrawal treatment, and did not improve predictive ability for levels of salivary miR-149. Given the sample size of infants with POE (n = 28), this analysis was likely underpowered to detect an effect. Of note, we have previously shown in a large cohort of infants (n = 121) that household income < USD 25,000 and public health insurance increased the likelihood of neurodevelopmental concerns at age 18 months, though neither modulated levels of a neurodevelopmental-associated microRNA in infant saliva. Nonetheless, future studies should repeat measurement of opioid-responsive miRNAs in a larger cohort and assess their relationship to medical/demographic variables. Such studies may also wish to assess the exosomal origins of salivary miRNA. Such information may help to refine neuron- and glia-related markers of NOWS, and optimize translational relevance.

In conclusion, this study identified a novel role for miR-149-3p in the attenuation of cell type proportion differences after neural progenitor differentiation during chronic morphine exposure. This effect is independent of *DLK1* clearance in the AGO2 complex. These findings may serve as a foundation for investigating the regulatory role of microRNAs in neuronal maturation and cell fate commitments during chronic opioid exposure and withdrawal. They also establish miR-149-3p as a possible relevant biomarker for infants with POE and NOWS.

## Supporting information

S1 Table Sequences of custom primers/microRNA mimics and all data. (XLSX)

S1 Fig Transfection efficiency has wider variability at higher concentrations of miRNA mimic. Total RNA was extracted from hiPSC-derived neural progenitors and levels of miR-149-3p was measured by RT-qPCR. Standard deviations of transfection efficiencies were larger with higher levels of miRNA mimic.
(TIF)

S2 Fig Time course of microRNA expression changes after morphine withdrawal and associated miR-149-3p putative transcript level changes. There were no significant differences in levels of candidate microRNAs 5 hours after morphine withdrawal (Two way ANOVA, F (6, 24) = 0.09, p = 0.99) (a). MicroRNA expression was measured over a time course of 12, 24, 48, and 96 hours after morphine withdrawal, with the most observed variation occurring 24 hours after morphine withdrawal (b-e). On DIV11, there were no significant differences in levels of let-7a-5p (One way ANOVA, F = 3.061, p = 0.084) (f) and miR-192-5p (One way ANOVA, F = 0.13, p = 0.878) (g) across treatment conditions. Also on DIV11, levels of SYNGAP1 were unchanged across treatment conditions (One way ANOVA, F = 0.49, p = 0.62) (h). SNORD44 was used as an internal control gene. All samples were repeated in technical triplicate for qPCR.
(TIF)

S3 Fig Inconsistent amplification of PRKCA and MECP2 transcript levels in AGO2-enriched pulldown samples. RT-qPCR was performed on AGO2-enriched immunoprecipitated RNA to assess for potential bound transcripts. There was minimal amplification of PRKCA (top), and inconsistent amplification of MECP2 (b) across treatment conditions.
(TIF)
