# Supplementary figures and images for: Overexpression of miR-149 attenuates opioid-related perturbations in neural stem cell fates and serves as a translational biomarker for infants with prenatal opioid exposure
Source: PLoS One. 2026 Mar 31;21(3):e0345640. doi: 10.1371/journal.pone.0345640 (PMC13038007; doi:10.1371/journal.pone.0345640)

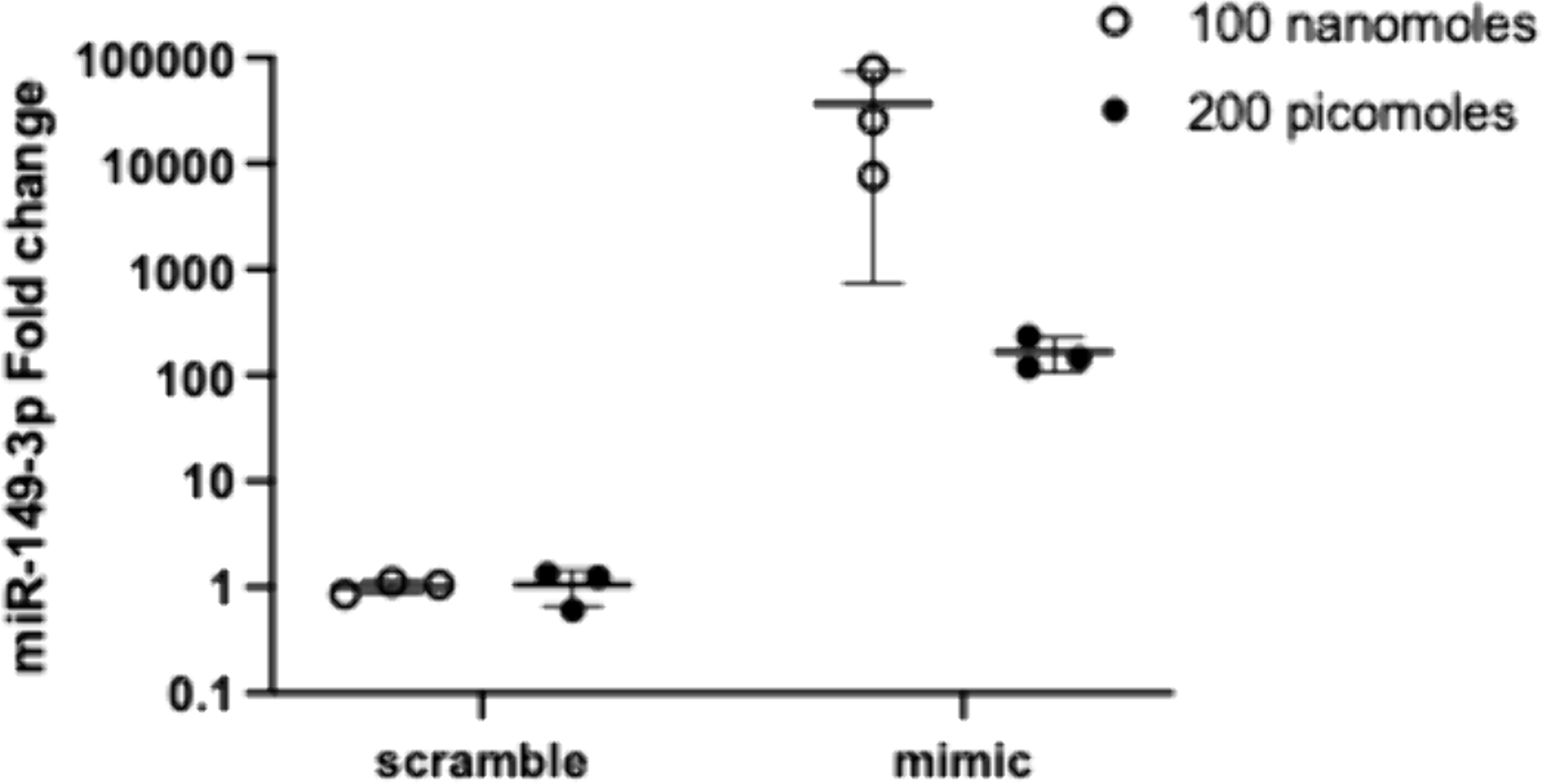

Supplement: S1 Fig — Total RNA was extracted from hiPSC-derived neural progenitors and levels of miR-149-3p was measured by RT-qPCR. Standard deviations of transfection efficiencies were larger with higher levels of miRNA mimic. (TIF) [file pone.0345640.s002.tif]

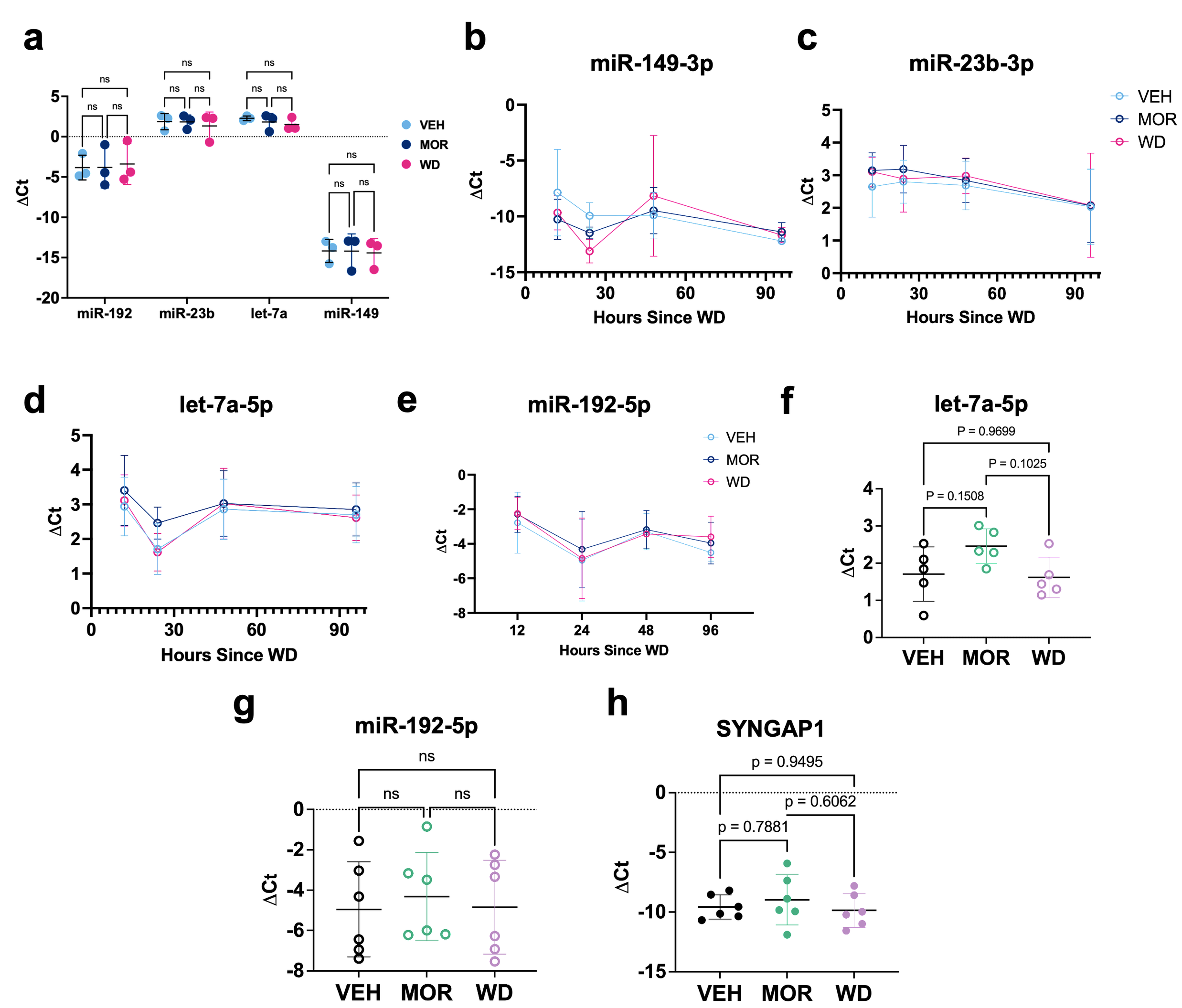

Supplement: S2 Fig — There were no significant differences in levels of candidate microRNAs 5 hours after morphine withdrawal (Two way ANOVA, F (6, 24) = 0.09, p = 0.99) (a). MicroRNA expression was measured over a time course of 12, 24, 48, and 96 hours after morphine withdrawal, with the most observed variation occurring 24 hours after morphine withdrawal (b-e). On DIV11, there were no significant differences in levels of let-7a-5p (One way ANOVA, F = 3.061, p = 0.084) (f) and miR-192-5p (One way ANOVA, F = 0.13, p = 0.878) (g) across treatment conditions. Also on DIV11, levels of SYNGAP1 were unchanged across treatment conditions (One way ANOVA, F = 0.49, p = 0.62) (h). SNORD44 was used as an internal control gene. All samples were repeated in technical triplicate for qPCR. (TIF) [file pone.0345640.s003.tif]

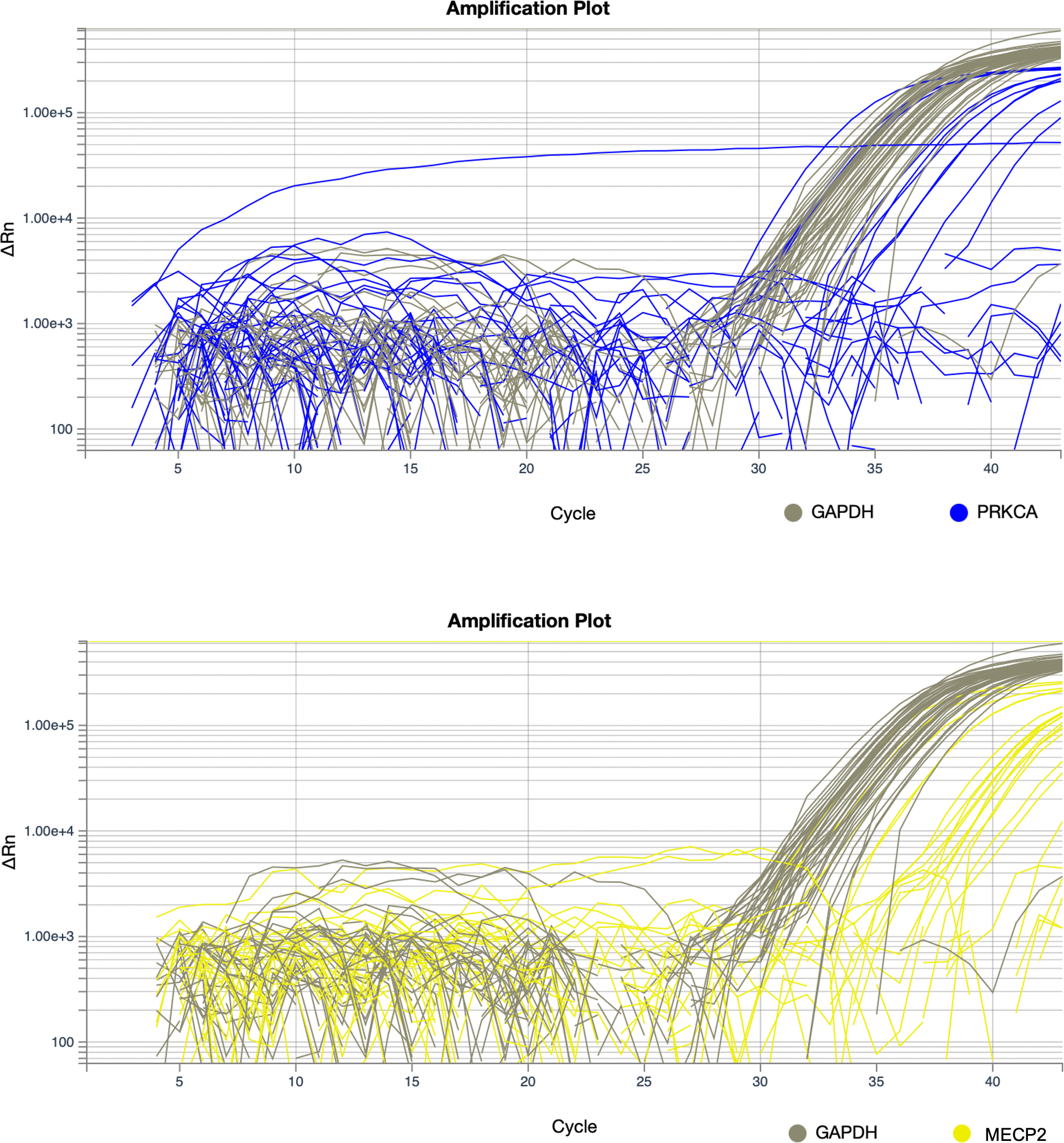

Supplement: S3 Fig — RT-qPCR was performed on AGO2-enriched immunoprecipitated RNA to assess for potential bound transcripts. There was minimal amplification of PRKCA (top), and inconsistent amplification of MECP2 (b) across treatment conditions. (TIF) [file pone.0345640.s004.tif]
